# Are Autobiographical Memories Inherently Social? Evidence from an fMRI Study

**DOI:** 10.1371/journal.pone.0045089

**Published:** 2012-09-21

**Authors:** Linda Wilbers, Lorena Deuker, Juergen Fell, Nikolai Axmacher

**Affiliations:** 1 Department of Epileptology, University of Bonn, Bonn, Germany; 2 German Center for Neurodegenerative Diseases (DZNE), Bonn, Germany; University College London, United Kingdom

## Abstract

The story of our lifetime – our narrative self – is constructed from our autobiographical memories. A central claim of social psychology is that this narrative self is inherently social: When we construct our lives, we do so in a real or imagined interaction. This predicts that self-referential processes which are involved in recall of autobiographical memories overlap with processes involved in social interactions. Indeed, previous functional MRI studies indicate that regions in the medial prefrontal cortex (mPFC) are activated during autobiographical memory recall and virtual communication. However, no fMRI study has investigated recall of autobiographical memories in a real-life interaction. We developed a novel paradigm in which participants overtly reported self-related and other-related memories to an experimenter, whose non-verbal reactions were being filmed and online displayed to the participants in the scanner. We found that recall of autobiographical vs. non-autobiographical memories was associated with activation of the mPFC, as was recall in the social as compared to a non-social control condition; however, both contrasts involved different non-overlapping regions within the mPFC. These results indicate that self-referential processes involved in autobiographical memory recall are different from processes supporting social interactions, and argue against the hypothesis that autobiographical memories are inherently social.

## Introduction

Episodic memory is defined as memory for events which can be exactly localized in space and time, and which can be retrieved by a “mental time travel” [1]. Typically, these events concern personal, autobiographical experiences (in the following, the terms “personal memory” and “autobiographical memory” are used interchangeably). If they are sufficiently relevant, they are repeatedly recalled, which usually involves considerable modifications of the initial experiences [2]–[5]. Therefore, the recall of autobiographical memories is a prototypical example of a memory reconstruction: It requires not just that memories be reliably stored and afterwards retrieved in identical form, but rather that they be integrated with self-referential processes to preserve a sense of being a coherent person over time (e.g., [6], [7]). As a result, recall of personal memories transforms experiences into a personal identity, the *autobiographical self* [8] or *narrative self* (e.g., [9]): Over time, your experiences define how you see yourself, and how you present yourself towards others – they become the story you tell about yourself.

Autobiographical memory encompasses both memory for facts about one's life (semantic autobiographical memory) and memory for specific episodes localized in time and space (episodic autobiographical memory). Here, we focus on episodic autobiographical memory because it has been argued that the autobiographical self is constructed of narratives [9], [10], and episodes putatively have a more narrative structure than semantic facts have.

Autobiographical memory recall is intimately linked to social interactions. First, autobiographical memory may support social functions in a very obvious way by establishing and maintaining social contacts and relationships. In fact, personal memories are often recalled in social situations – a conversation appears more reliable, convincing and intimate when personal memories are being exchanged [3]. Second, according to theories in social psychology, autobiographical memory recall must also be considered social on a more fundamental level, because a person's view of himself or herself results from real or imagined interactions with other persons. Thus, according to this view, autobiographical memory recall is inherently social as it implies a report of personal experiences to a fictitious (or real) addressee [2], [10].

Several studies have investigated the neural correlates of episodic autobiographical memory recall and have found that it involves episodic memory processes associated with the medial temporal lobe (e.g., [11]–[13]) as well as self-referential processes [14]–[17]. The latter ones have been associated with activity within the medial prefrontal cortex (mPFC) [14], [17], [18]: For example, it was shown that the mPFC is more active while watching self-taken photos in comparison to photos taken by someone else [14]. Other functional MRI studies support the idea that these self-referential processes are actually linked to social interactions. First, the mPFC is also activated in “Theory of Mind” paradigms involving the attribution of mental states, beliefs, and knowledge to other people [19], [20]. Second, even more direct support for a role of the mPFC in social interactions has been found in virtual interaction paradigms in which participants imagined to talk to their friends [21] or to other persons in unstable social hierarchies [22]. These studies suggest that the narrative self – which is being constructed by autobiographical memory recall – is actually intimately linked to social interactions.

Studying the social nature of the narrative self requires one to contrast recall of memories that are integrated into the self with recall of memories that do not become part of the self. We therefore used a paradigm in which participants either recalled personal experiences or fictitious events from books or movies (e.g., Harry Potter's first day in his new school in Hogwarts). In both cases, memories can be localized in space and time, but only the personal events can be integrated with self-referential processes and become part of the narrative self. Furthermore, to study the social character of these self-referential processes, we contrasted a social condition (in which memories were reported in a live interaction to the experimenter, who reacted appropriately) with a non-social condition, in which memories were loudly described as well, but only the video of an animated face was shown. We hypothesized that if the self-referential processes involved in autobiographical memory recall are indeed inherently social, recall of personal as compared to fictitious events should be associated with activation of the same mPFC region which is engaged in the social as compared to the nonsocial task – even if no overt social interaction takes place, i.e., even in the nonsocial condition. If, on the other hand, social interactions and autobiographical memories independently affect the self, different mPFC subregions should be activated in these two contrasts. More specifically, we wanted to test whether a contrast of autobiographical vs. non-autobiographical episodic memory (collapsed across the social and the non-social condition) reveals activation in the same mPFC subregions as the contrast of social vs. non-social recall (collapsed across the autobiographical vs. non-autobiographical condition).

## Materials and Methods

### Ethics statement

The study was approved by the local medical ethics committee (“Ethikkommission an der Medizinischen Fakultaet der Rheinischen Friedrich-Wilhelms-Universitaet Bonn”), was according to the latest version of the Declaration of Helsinki, and all subjects provided written informed consent.

**Figure 1:**
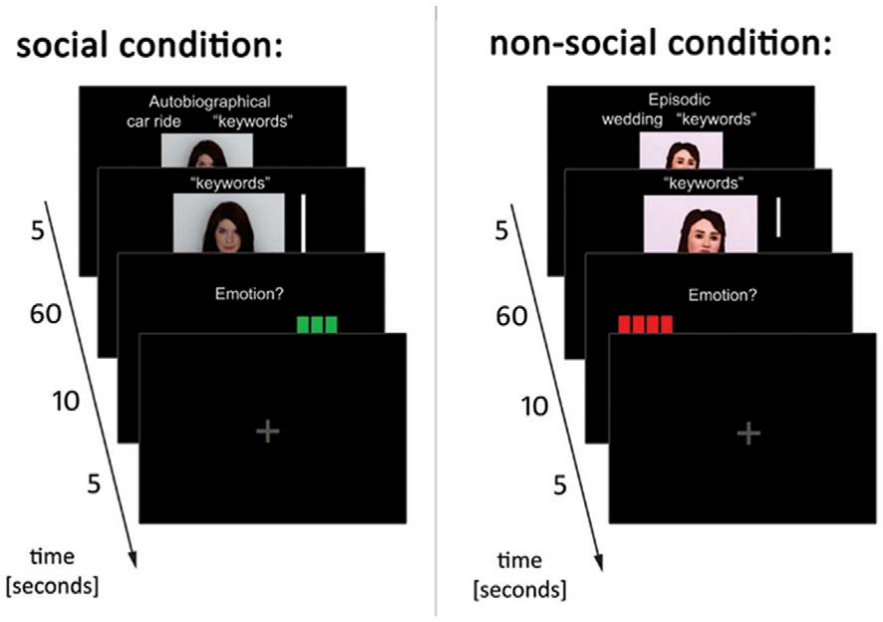
Experimental paradigm in the social and non-social condition. **Left:** Social condition. Within the scanner, the participant was presented with the topic and the matching key word and asked to recall his/her memory loudly while the investigator was listening. The investigator's reaction to the story was filmed by a webcam and was online back-transferred to the participant in the scanner, creating a real live interaction between the participant and the experimenter. **Right:** Non-social condition. Participants were presented with the topic and the matching key word and were asked to recall the respective memories loudly without the experimenter listening to it. Subjects were presented videos of an avatar that showed non-verbal reactions, which, however, were not synchronized to the participant's report, resulting in no social interaction.

The individual depicted on Figure 1 (the first author, LW) has given written informed consent (as outlined in the PLoS consent form) to publish this picture.

### Subjects

Thirty (19 female, 11 male) healthy, right-handed, native German speakers with normal or corrected-to-normal vision participated in the experiment (mean age: 23.2 years, range: 20–26 years). They were recruited from the University of Bonn as well as via internet advertisement. The study was approved by the local medical ethics committee, and all subjects gave written informed consent.

### Experimental Paradigm

The paradigm consisted of two sessions that took place on two consecutive days. On the first day, participants had to go through a computer program and recall 32 episodic memories that they could localize in space and time. From a total list of 35 possible topics, participants could choose those 16 topics for which they were able to remember one personal and one fictitious event (e.g., “meeting an important person/partner”, embarrassing event”, “car ride”, “sport event”). For each of these topics, they were asked to think of one personal and one fictitious event that matched each of these topics (resulting in 32 events in total). A complete list of the topics is presented in Table S1. *Personal memories* were defined as events that had been experienced by the participants themselves and could thus become part of the narrative self of a person. In contrast, *fictitious memories* were defined as memories for events originating from movies or books that could therefore not become part of the narrative self. Although we could not exclude that recall of these fictitious events included recall of the autobiographical situations in which they were initially acquired (e.g., reading a book or watching a movie), we reasoned that it still makes a relevant difference whether some event has happened to oneself or to, e.g., Harry Potter. For each selected event, participants were instructed to write down a key word (allowing them to remember it later) as well as to rate its emotional valence (from −5, very negative, to +5, very positive), vividness (from 0, very vague up to 5, vivid) and the age of the memory in categories (last week, last month, last year, older than 12 month). Key words were chosen by the participants to denote both the fictitious episodes and the autobiographical episodes. Participants were instructed to select a word which should allow them to afterwards easily recall this episode. For example, to the topic “car accident”, one participant noted “L.A. crash, argument way home” for a fictitious event (obviously from the movie “L.A. crash”) and “Betty [name changed to ensure privacy], Mercedes, first crash” for the autobiographical event. Participants were asked to balance the age of the autobiographical and episodic memories – after completing the questionnaire, the mean age of the episodic autobiographical memory had to be as balanced as possible. In addition, participants were instructed that the memories should meet the following criteria. First, all memories should be salient and involve some emotional engagement, but memories should not be related to a traumatic event (such as the death of a close relative). Second, memories should be specific, i.e., they should refer to one specific event instead of a general or repeated situation.

During the second session on the subsequent day, participants loudly recalled their memories during fMRI scanning using an MRI-compatible microphone. We will first give an overview of the experiment and then provide details. Prior to scanning, all subjects were instructed by presenting them the exact design of the experimental paradigm outside of the scanner. We ensured that all participants had understood their task by explaining it to them extensively and having them repeat it in their own words. *Brief description:* Recall occurred either in a social or in a non-social condition. In the *social condition* (Fig. 1A), the experimenter (LW; outside of the scanner) listened to the narrative and was simultaneously filmed by a webcam (Logitech® Webcam Pro 9000; Logitech®, Morges, Switzerland). The online video of the experimenter's non-verbal reaction (such as nodding, laughing/looking serious, raising the eyebrows, tilting the head etc.) was transferred to the participant's monitor inside the scanner, resulting in a live social interaction between the participant reporting the memory and the experimenter listening to it. The experimenter showed an adequate reaction to the participant's narratives. In order to reduce variability between the different episodes and to increase experimental control, the experimenter did not react verbally. In the *non-social condition* (Fig. 1B), the experimenter did not listen to the participant's report. The experimenter was not filmed, and no social interaction occurred. To ensure similar visual input, participants were presented videos of a virtual person (an “avatar”) that showed different random non-verbal facial expressions (e.g. laughing, serious face expression etc.) but was not reacting adequately to the participant's report. We decided to use a computer animated Avatar instead of a human who did not respond adequately as the latter option might have irritated the participants – a human being who either does not respond at all or responds inadequately could still have been perceived as social. Presentation of the Avatar excluded that participants believed there was any real person they interacted with, because they were clearly instructed that whenever an Avatar was presented, no one would be listening to their stories. Avatars were created using “the Sims 3” (Electronic Arts GmbH). In both the social and the non-social condition, 8 personal and 8 fictitious memories were recalled, resulting in 32 trials that were presented in a randomized order.

*Detailed description*: In both the social and the non-social conditions, the key words which had been chosen by the participants were presented via MRI-compatible goggles (Nordic Neuro Lab, Bergen, Norway) using Python software. First, a screen was shown for 5 s indicating a topic, the matching key words and a photo of either the avatar or the experimenter (indicating that either a social or a non-social trial would follow). During the following memory recall, participants had to report loudly their memory corresponding to the key word. An MRI-compatible microphone was used, allowing the experimenter to listen to the narrative (Fibersound® Microphone Model FOM1-MR and Fibersound® Control Model FOM1-DRx Battery/wall powered; Micro Optics Technologies Fibersound™ Audio, Middleton, USA). Each recall lasted 60 s, during which a white bar on the right side of the screen indicated the remaining time for each narrative to the participant. This was followed by an interrogation about the current emotional state of the participant (emotional valence from −5, very negative, to +5, very positive) that lasted 10 s, to which participants responded by button presses. A pause of 5 s followed, during which a fixation cross was shown. In order to prevent movement during overt speech, we fixed the participants very well by using pads in order to stabilize the head. In addition, a strip of plaster was adhered over their chin which provided the participants with sensory feedback when they moved their head. Only participants with a movement of less than the diameter of one voxel (3 mm) were included in subsequent analysis. Figure S1 indicates the movement parameters of all 17 included participants.

### MRI data acquisition

Thirty-seven axial slices were collected at 3T (Trio, Siemens, Erlangen, Germany). We collected T2*-weighted, gradient echo EPI scans (slice thickness: 2.5 mm; matrix size: 64×64; field of view: 210×210 mm; repetition time: 2500 ms; echo time: 35 ms). Thereafter, we acquired a 3D-sagittal T1-weighted MPRAGE sequence for each subject for anatomical localization (number of slices: 160; slice thickness: 1 mm; inter-slice gap: 0.5 mm; voxel size: 1×1×1; matrix size 256×256; field of view: 256 mm; echo time: 3.42 ms; repetition time: 1570 ms).

### Data analysis

MRIs were pre-processed in SPM 5 (www.fil.ion.ucl.ac.uk/spm/) using standard pre-processing steps including realignment, unwarping, normalization, and smoothing with a 6-mm Gaussian kernel. Pre-processed data were fitted by the convolution of multiple regressors with a canonical hemodynamic response function to obtain parameter estimates for each condition covariate. The following set of regressors was used: four regressors for the different conditions (1: personal social; 2: personal non-social; 3: fictitious social; 4: fictitious non-social), one regressor for the screen showing the topic and keywords, one for the emotional interrogation. Movement was modeled with a set of six continuous regressors. We used the following contrasts: (A) “social vs. non-social”: (regressor 1+regressor 3)>(regressor 2+regressor 4); (B) “autobiographical vs. non-autobiographical”: (regressor 1+regressor 2)>(regressor 3+regressor 4). In addition, we ran an alternative model including regressors for parametric modulation of the four experimental conditions by emotional content during scanning. Results from these analyses were almost identical to the results from the analysis without parametric modulation and are shown as Figure S2. All figures with fMRI results are displayed using neurological convention (left hemisphere on the left side of the figure). To identify significant activations, we used a voxel threshold of P<0.05, corrected for multiple comparisons using the FDR procedure of SPM5, and at least 5 contiguous voxels.

### Overlap analysis

Visually, it appeared that activity during the autobiographical vs. non-autobiographical condition did not overlap with activity during the social vs. non-social condition. However, the amount of overlap depends on the statistical threshold used and is thus basically conventional. Therefore, we developed a non-parametric surrogate-based permutation approach to test if the amount of overlap observed in the empirical data exceeds the amount of overlap that would have been expected by chance given the spatial correlation structure of the empirical data. The common approach in testing if two regions of activation are overlapping would be to apply a conjunction analysis [23]. However, here we wanted to test if the two areas of activation did not overlap beyond what would be expected by chance. The question at hand therefore was: given the amount of activation (number of significant voxels) for the two conditions, and taking into account the three-dimensional layout of the MRI data, how likely is it to get a certain amount of overlap? And more specifically, is the amount of overlap that we find in the actual data significantly greater than what would be expected by chance? The most parsimonious approach to test this would have been to calculate a binomial statistics taking into account the overall number of voxels in the brain as well as the number of voxels activated in each contrast and to compute whether the observed number of overlapping voxels is larger than the expected number of overlapping voxels. However, this simple approach does not take into account that the empirical data show a high degree of spatial correlations, which likely influences the expected overlap between contrasts. Therefore, we designed a bootstrapping approach based on surrogate data with the same spatial correlation structure as in the empirical data. In detail, we first created surrogate second-level significance maps. We used SPM5 to calculate a second-level analysis across the 17 participants, but either with the empirical contrast (e.g. social vs. non-social) or the inverse contrast (e.g. non-social vs. social), randomly selected for each participant. Then we applied a group-level t-test against zero to these 17 contrast images. As a result, we obtained significance maps with t-values for every voxel that was included in the SPM5 brain mask. This procedure was done for both social vs. non-social and for the autobiographical vs. non-autobiographical contrast, and was performed a hundred times each. In each of the resulting 100 surrogate significance maps, we adjusted the significance threshold such that the number of “significant” voxels in each contrast matched the number of significant voxels found in our real data. This was done separately for the two main contrasts (social vs. non-social and autobiographical vs. non-autobiographical). Finally, we calculated the overlap between the pairs of thresholded significance maps using inclusive masking and thus obtained a surrogate distribution of the amount of overlap expected by chance.

The surrogate statistics was conducted using SPM5 (to calculate the contrast images for each participant) as well as custom code (to randomly draw either a contrast or its reverse in each participant, to perform the group analysis based on t-tests in each voxel, and to determine the level of overlap in the resulting group-level images).

## Results

### Behavioral results

On the first day, participants rated 32 episodic memories according to their emotional value, vividness, and age. There were no significant differences between the autobiographical and non-autobiographical memories in terms of the level of emotion (autobiographical: 1.24±0.26 [mean ± s.e.m.]; non-autobiographical: 0.81±0.25; t_16_ = −1.67; p = 0.11) and level of age (repeated t-tests in the different age groups: all t_16_<0.51; all p>0.52). The only significant difference between autobiographical and non-autobiographical memories was the level of detail (autobiographical: 3.52±0.16; non-autobiographical: 2.39±0.18; t_16_ = 6.12; p<0.0001). During the fMRI scan, participants were interrogated about their current emotion after reporting each event. A two-way ANOVA with “social vs. nonsocial” and “autobiographical vs. non-autobiographical” as repeated measures revealed that memory recall in the social condition was associated with significantly more positive emotions as compared to the non-social condition (F1,16 = 4.98; p = 0.04), while there was no difference between the autobiographical as compared to the non-autobiographical condition (F1,16 = 2.92; p = 0.11) and no interaction (F1,16 = 0.69; p = 0.42).

### FMRI results

**Figure 2:**
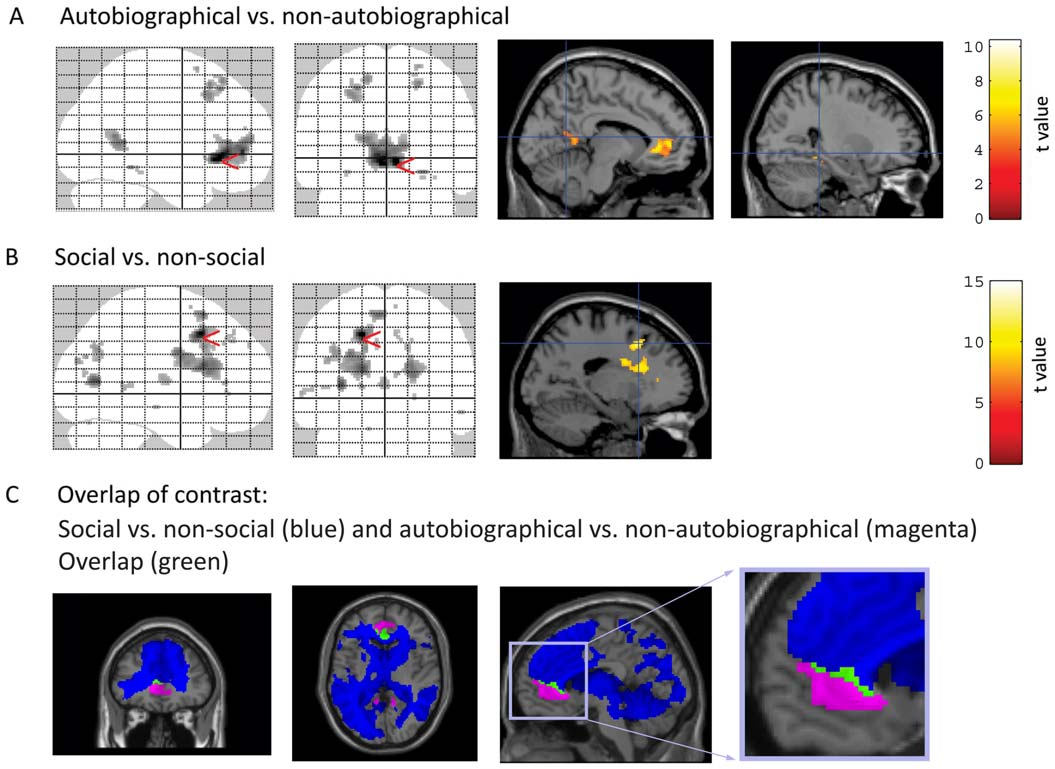
Neural activations related to autobiographical memory and social interactions. **A:** Autobiographical vs. non-autobiographical memory recall (for graphical depiction, we chose a threshold of p_FDR_<0.05). **B:** Social vs. non-social (for graphical depiction, we chose a threshold of p_FWE_<0.05). Activation can be seen in the dorsal part of the mPFC, cuneus, precuneus, and in the temporoparietal junction. **C:** Overlap of the contrasts social vs. non-social and autobiographical vs. non-autobiographical (identical threshold of p_FDR_<0.05). Blue indicates the social vs. non-social contrast, magenta the autobiographical vs. non-autobiographical contrast and green the overlap.

First, we assessed the main effect of autobiographical memory (contrast of autobiographical with non-autobiographical trials). Retrieval of autobiographical memories was associated with increased activation of a medial prefrontal region corresponding to the pre- and subgenual mPFC (Fig. 2A, C) as well as the bilateral cuneus and the bilateral parahippocampal cortex.

Second, we identified the main effect of social interactions (contrasting social versus non-social trials). This contrast revealed significant activation increases in widespread brain regions (see Table 1) including a relatively dorsal medial prefrontal cluster (see Fig. 2A, B). This contrast yielded similar results in the autobiographical and in the non-autobiographical condition when considered separately: The interaction contrast does not reveal any super-threshold voxels for this contrast (or for the reverse contrast).

**Table 1:** Overview of all significantly activated regions.

MNI coordinates				
x	y	z	t value	
**social vs. non-social**				
−18	16	46	14.91	supragenual mPFC
−32	−68	10	11.39	middle occipital gyrus
−16	18	22	11.32	supragenual mPFC
42	−86	−6	2.99	inferior occipital gyrus
16	−98	−2	2.93	inferior occipital gyrus
22	−78	46	2.93	precuneus
12	−80	48	2.31	precuneus
38	−68	36	2.82	precuneus
16	−60	6	2.23	inferior occipital gyrus
**autobiographic vs. non-autobiographic**				
6	30	−2	10.32	subgenual mPFC
−2	26	−4	9.34	subgenual mPFC
−6	48	2	8.14	pregenual mPFC
−14	−54	14	6.12	lingual gyrus
−10	−50	8	6.04	cuneus
−22	30	50	5.92	superior frontal gyrus
12	−52	8	5.73	cuneus
8	−46	4	4.49	cuneus
24	22	54	5.70	superior frontal gyrus
−26	22	46	5.63	superior frontal gyrus
−14	32	56	5.61	superior frontal gyrus
28	−40	−10	5.56	parahippocampal cortex
24	12	66	5.04	superior frontal gyrus

Activations were thresholded at a voxel-wise threshold of p_FDR_<0.05 and a cluster threshold of at least 5 contiguous voxels.

Third, we wondered whether those brain regions that are recruited during autobiographical as compared to non-autobiographical memory recall are also activated during social interactions. Figure 2C allows one to directly compare activations related to the two main effects. We found that subregions of the mPFC are either activated for autobiographical memory or social interactions, and lie next to each other but with virtually no overlap: For the autobiographical vs. non-autobiographical memory contrast, there was activation of the pre- and subgenual mPFC, while the social vs. nonsocial contrast was associated with activation of the supragenual mPFC extending into dorsal midline regions. The number of overlapping voxels activated during both contrasts is only around 5.5% of all voxels activated in either contrast (we calculated the overlapping voxels by taking the number of voxels of overlap and dividing it by the sum of voxels activated in either contrast). Furthermore, we explored if the amount of overlap between the two contrasts exceeded the amount of overlap expected by chance. Using a non-parametric surrogate-based permutation approach (see Methods), we found that the amount of overlap in the empirical data did not exceed the 95^th^ percentile of the surrogate distribution (it was in the 86^th^ percentile) and was thus not significantly greater than what would be expected by chance.

In order to exclude that our results were biased by the significantly more positive emotion ratings in the social as compared to the non-social condition, we conducted an additional general linear model with additional regressors for a parametric modulation of activity by emotional ratings. As shown in the Figure S2 and Table S2, results were almost identical to our previous model. Notably, this analysis was not performed to investigate the effect of emotion on brain activation patterns (this was not among the aims of our study), but only in order to exclude a bias in the results from the contrast of the main (non-modulated) regressors.

Finally, we tested whether our results depended on gender differences. However, a two-sample t-test (with “gender” as group variable) revealed no significant group differences, neither for the contrast of autobiographical vs. non-autobiographical trials nor for the contrast of social vs. non-social trials.

## Discussion

In this study, we used a new paradigm involving a real social interaction to investigate whether regions associated with the narrative self constructed by autobiographical memories overlap with regions activated during social interactions. In other words, we studied neural activation patterns underlying autobiographical memory and social interactions, and analyzed whether these processes share a common pattern of activation in the mPFC. However, there was virtually no overlap between the brain regions corresponding to these two processes, as confirmed by a non-parametric surrogate statistics.

Former studies dealing with social interactions concentrated on different aspects of them. Sassa and colleagues [21] investigated the effect of communicative speech. Participants engaged in an overt communication and imagined either to talk to an actor or to verbally describe a situation while observing video clips of an action performed by an actor in a typical daily situation. Higher activation was observed during communication than during description in polar and dorsal regions of the mPFC, in the bilateral anterior superior temporal sulci, and in the left temporoparietal junction. These regions closely resembled those observed in our contrast of social vs. non-social trials. This study investigated the effect of social communication, but did not involve a live social interaction. A live face-to-face interaction during fMRI scanning was for the first time implemented by Redcay and coworkers [24]. In this study, participants interacted with the experimenter in a two simple cooperative games. Again, they found (among other regions) activation of the dorsal mPFC (corresponding to the supragenual anterior cingulate cortex). This design involved a real social interaction between participants and experimenter, but was still limited in that the social exchange was simple, heavily scripted, and very predictable. One crucial part of real-life communication is self-expression, which makes a conversation more individual. In our paradigm, participants engaged with the experimenter in a conversation about salient life events. Although autobiographical memory has been investigated in previous studies, the real-live interaction established in our paradigm was actually new. We believe that this experimental approach can be used to address a variety of topics in social neuroscience.

In the social as compared to the non-social trials, we found significant activations of widespread networks including a dorsal subregion of the mPFC. These results are consistent with previous studies on social interactions [21], [22], [24]. In contrast, retrieval of autobiographical, personal memories – as compared to recall of fictitious events – was associated with significantly enhanced activations of a ventral subregion of the mPFC, as well as bilateral cuneus and parahippocampal cortices.

A meta-analysis on 24 functional imaging studies identified several regions commonly activated during autobiographical memory tasks, which included the mPFC, the retrosplenial/posterior cingulate cortices (closely adjacent to the cuneus) and the medial temporal lobe [25]. These regions are very similar to those in which we observed increased activation during the autobiographical vs. non-autobiographical episodic memory recall. Furthermore, our results are consistent with previous studies on the neural basis of self-referential processing during autobiographical memory. For example, there was increased activation of the ventromedial PFC, the cuneus and the parahippocampal gyrus during viewing of self-taken photographs as compared to photographs taken by other participants [14].

In our study, we did not focus on semantic autobiographical memory. Therefore, from the present results we cannot draw direct inferences about the interaction between semantic autobiographical memory processes and social interactions. A previous fMRI study attempting to dissociate the neural basis of episodic and semantic autobiographical memory reported similar activations in the anterior mPFC, however more pronounced in the episodic autobiographical memory condition [26]. Based on these and the present results, one may speculate that also the functional activations associated with semantic autobiographical memory processes are distinct from activations during social interactions, although this remains to be tested.

We used a control condition (the avatar) that was designed to share as many perceptual properties with the real social interaction condition as possible without involving any real interaction. However, it should be noted that our results in the social vs. non-social contrast might be affected by other factors as well – for example, by the fact that adequate feedback was only given in one condition but not in the other, that the facial reaction of the avatar were more stereotypical etc. However, we do not think that our main findings can only be explained by these potential biases: First, it can be argued that these differences are inherent in the fact that the relatively complex concept of “social interaction” should only apply to the real interaction condition but not to the control condition. Second, even if the social vs. non-social contrast did not selectively isolate social interactions but also involved other factors (as might be suggested by the fact that we found very wide-spread activations in this contrast), one would rather overestimate any overlap with the contrast of autobiographical vs. non-autobiographical memories. In other words, a more selective contrast would have resulted in less activation and even less overlap.

As our hypotheses were specifically related to the mPFC, we will concentrate on this region in the remainder of the Discussion. In particular, we aimed to investigate whether there is a strong degree of overlap in the neural activations within the mPFC related to social interactions and autobiographical memory. The mPFC supports self-referential processes (e.g., [27]) during both social interactions [20], [21] and autobiographical memory [14], [17]. Therefore, finding similar activations during social interactions and autobiographical memory would have been consistent with the view that the same self-referential processes are recruited during these two processes. In other words, it would support the theory that the narrative self that is constructed by recall of personal memories is inherently social [2], [10]. However, we found that autobiographical memory recall and social interactions were associated with distinct, mutually exclusive mPFC subregions. This suggests that different self-referential processes occur during autobiographical memory and social interactions.

Only few previous studies addressed the question whether self-referential processes employed during autobiographical memory and social interactions differ or not. Northoff et al. [27] re-analyzed previous neuroimaging studies related to distinct concepts of the self and concluded that wide-spread networks within cortical midline structures (including regions of the mPFC) are active across a large range of studies on self-referential processes. This suggests that there is no clear-cut difference between mPFC regions (and self-referential processes) during social interaction and autobiographical memory. Similarly, Saxe et al. [20] studied the relationship of brain regions active during a theory of mind task (involving social interaction) and a self-reflection task. Regions of overlap were found in the mPFC as well as in the precuneus. However, none of these studies involved a real live interaction as our paradigm did, which might explain the differential results.

Now, if the narrative self is not inherently social, how may it be affected by social interactions? Saxe and colleagues [20] introduced six different models of how the processes of self-reflection, autobiographical memory and theory of mind could interact with each other. These models differ in the degree of overlap and the causal interactions between these processes. Their model 4 would fit best to our results. According to this model, self-referential processes and autobiographical memory overlap as they both depend on a conception of a stable and coherent self. However, this model is consistent with the idea that there are different aspects of the self related to either personal experiences or interactions with others.

One limitation of our study is that one might ask if we actually investigated personal memories that had an impact on the participants' personality, in the way that they became part of his or her narrative self. Our selection of topics aimed to capture typical relevant life events, and participants were instructed to select personally salient experiences. However, the actual personal relevance of these memories for the construction of the self remains unknown (and is difficult to assess in typical study participants). Future studies with other populations of participants should aim to further elucidate this issue. For example, relevant life events could be assessed in psychologists undergoing training in psychotherapy.

Another aspect that could be improved in future investigations is the social interaction. Although there was an actual live interaction, the experimenter in our study did not ask questions herself (to ensure higher experimental control), but it would be interesting to investigate the effects of an actual conversation on neural activation patterns.

While the contrast analyses were conducted using parametric statistics, the overlap analysis was done using a non-parametric approach. It should be noted that these two analyses make different assumptions concerning the distribution of the BOLD signal (normal distribution is assumed for parametric but not for non-parametric analyses). Parametric analyses are more commonly used for fMRI data and were employed for the main analyses. However, the issue whether there was more overlap in the data than would be expected by chance could not be tested parametrically (because the distribution of chance overlap is unknown). Therefore, we implemented a custom non-parametric analysis. Possibly, this analysis could be refined by matching the spatial correlation structure of the surrogate data more closely with the correlation structure of the real data.

A final limitation is due to the fact that in our group of participants, more female than male participants were scanned. As we used a within-subject design, it is unlikely that the main effects are driven by gender differences. Furthermore, a two-sample t-test did not reveal any significant influence of gender on our results.

## Conclusions

In this paper, we used fMRI during a novel experimental paradigm involving real social interactions to address the question whether the narrative self as constructed by episodic autobiographical memories is inherently social. Our results did not support this hypothesis but rather indicated that adjacent but non-overlapping regions support social interactions and autobiographical memories. They rather suggest a model in which social interactions are independently processed from self-related networks, but may subsequently shape self-referential processes.

## Supporting Information

Figure S1 **Movement of participants during scanning.** This figure shows movement parameters of all 17 participants which were finally included in the analysis of fMRI data. Each subpanel depicts the movement of one participant in the x, y, and z direction. Only subjects who moved less than the extent of one voxel (3 mm) met the inclusion criteria.
(TIF)

Figure S2 **Neural activations related to autobiographical memory and social interactions, when emotion ratings are included as additional regressors.** **A:** Autobiographical vs. non-autobiographical memory recall (thresholded at p_FDR_<0.05). **B:** Social vs. non-social (for graphical depiction, we chose a threshold of p_FWE_<0.05). The results are very similar to those of the alternative general linear model without inclusion of emotion ratings. **C:** Overlap (in orange) of the contrasts social vs. non-social (green) and autobiographical vs. non-autobiographical (red; identical threshold of p_FDR_<0.05).
(TIF)

Table S1 **List of topics.**
(DOCX)

Table S2 **Overview of all significantly activated regions when the rating of emotions is included as a regressor.** Activations were thresholded at a voxel-wise threshold of p_FDR_<0.05 and a cluster threshold of at least 5 contiguous voxels.
(DOCX)
