# Supplementary figures and images for: Are Autobiographical Memories Inherently Social? Evidence from an fMRI Study
Source: PLoS One. 2012 Sep 21;7(9):e45089. doi: 10.1371/journal.pone.0045089 (PMC3448611; doi:10.1371/journal.pone.0045089)

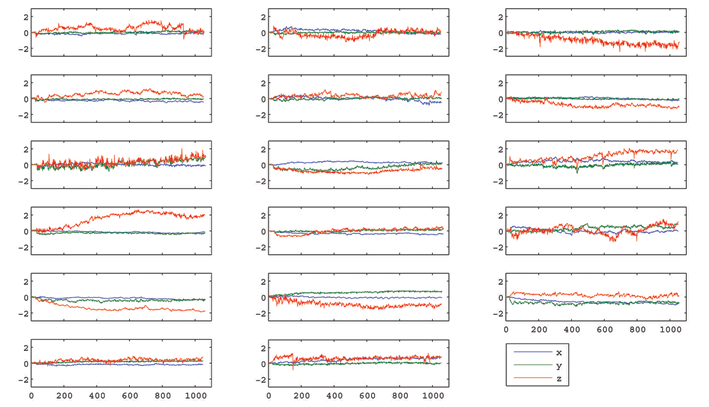

Supplement: Figure S1 — Movement of participants during scanning. This figure shows movement parameters of all 17 participants which were finally included in the analysis of fMRI data. Each subpanel depicts the movement of one participant in the x, y, and z direction. Only subjects who moved less than the extent of one voxel (3 mm) met the inclusion criteria. (TIF) [file pone.0045089.s001.tif]

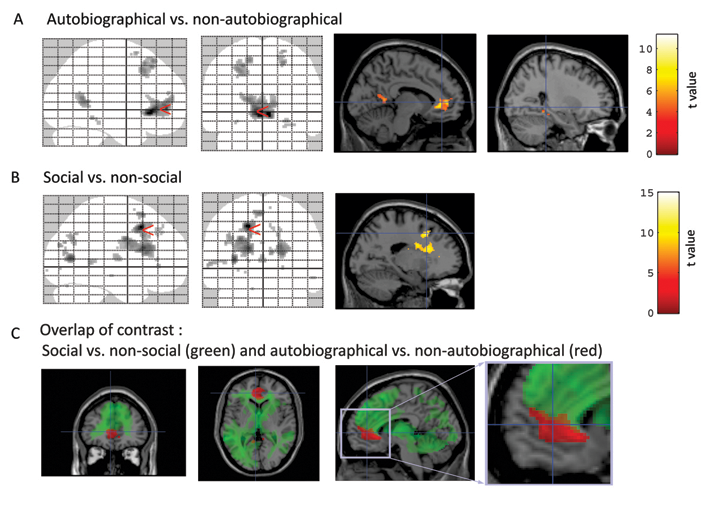

Supplement: Figure S2 — Neural activations related to autobiographical memory and social interactions, when emotion ratings are included as additional regressors. A: Autobiographical vs. non-autobiographical memory recall (thresholded at pFDR<0.05). B: Social vs. non-social (for graphical depiction, we chose a threshold of pFWE<0.05). The results are very similar to those of the alternative general linear model without inclusion of emotion ratings. C: Overlap (in orange) of the contrasts social vs. non-social (green) and autobiographical vs. non-autobiographical (red; identical threshold of pFDR<0.05). (TIF) [file pone.0045089.s002.tif]
