# Supplementary material for: Are Autobiographical Memories Inherently Social? Evidence from an fMRI Study
Source: PLoS One. 2012 Sep 21;7(9):e45089. doi: 10.1371/journal.pone.0045089 (PMC3448611; doi:10.1371/journal.pone.0045089)
Supplement: Table S1 — List of topics. (DOCX) [file pone.0045089.s003.docx]

| **List of Topics** |  |
| --- | --- |
|  |  |
| ***German:*** | ***English:*** |
| Eltern (positives Erlebnis) | Parents (positive event) |
| Eltern (negatives Erlebnis) | Parents (negative event) |
| Peinliches Erlebnis | Embarrassing event |
| Partner kennenlernen / wichtigen Menschen | Meeting the partner or an important person |
| Urlaub | Holiday event |
| Schönes Fest / Party | Party, festival |
| Krankheitsfall | Case of illness |
| Ausflug | Excursion |
| Freunde kennenlernen | Get to know friends |
| Museum / Theater (kulturelles Ereignis) | Museum / theater (cultural event) |
| Unfall | Accident |
| Restaurantbesuch | Going to a restaurant |
| Schönes Erlebnis mit Freunden | Nice experience with friends |
| Sporterlebnis | Sport event |
| Autofahrt/Erlebnis mit einem Auto | Car ride |
| Vorstellungsgespräch/1. Arbeitstag | Job interview / first day of work |
| Familienfeier | Family festival |
| Schwierige Prüfung/Herausforderung | Difficult exam, challenge |
| Ein Erlebnis aus der Schule | Event in school |
| Erlebnis mit einem Tier | Experience with an animal |
| Arztbesuch | Consultation |
| Geburtstagsfeier | Birthday party |
| Zoobesuch | Zoo visit |
| Hochzeit | Wedding |
| Hotel-/Barszene | Hotel /bar event |
| Kochereigneis | Cooking event |
| Spaziergang | Walk |
| Erlebnis mit einem Kind | Event with a child |
| Erlebnis mit unverschämter Person | Rude person |
| Missverständnis | Misunderstanding |
| Lustiges Erlebnis | Funny event |
| Wutausbruch | Outburst of fury |
| Geschwister (positives Erlebnis) | Brothers and sisters (positive event) |
| Geschwister (negatives Erlebnis) | Brothers and sisters (negative event) |
| Ein Erlebnis in einer Kirche | Event in a church |
