# Supplementary material for: Are Autobiographical Memories Inherently Social? Evidence from an fMRI Study
Source: PLoS One. 2012 Sep 21;7(9):e45089. doi: 10.1371/journal.pone.0045089 (PMC3448611; doi:10.1371/journal.pone.0045089)
Supplement: Table S2 — Overview of all significantly activated regions when the rating of emotions is included as a regressor. Activations were thresholded at a voxel-wise threshold of pFDR<0.05 and a cluster threshold of at least 5 contiguous voxels. (DOCX) [file pone.0045089.s004.docx]

| **MNI coordinates - Emotion** | | |  |  |
| --- | --- | --- | --- | --- |
| **x** | **y** | **z** | **t value** |  |
|  |  |  |  |  |
| **social vs. non-social** | |  |  |  |
|  |  |  |  |  |
| -18 | 16 | 46 | 14.95 | supragenual mPFC |
| -16 | 20 | 22 | 11.72 | supragenual mPFC |
| -32 | -68 | 10 | 11.69 | middle occipital gyrus |
| 22 | -78 | 46 | 3.01 | precuneus |
| 12 | -80 | 48 | 2.45 | precuneus |
| 28 | -78 | 40 | 2.38 | precuneus |
| 42 | -86 | -6 | 2.91 | inferior occipital gyrus |
| 16 | -60 | 4 | 2.33 | inferior occipital gyrus |
|  |  |  |  |  |
| **autobiographic vs. non-autobiographic** | | | |  |
|  |  |  |  |  |
| 6 | 30 | -2 | 11.29 | subgenual mPFC |
| -2 | 26 | -4 | 9.30 | subgenual mPFC |
| -6 | 48 | 2 | 8.22 | pregenual mPFC |
| -14 | -52 | 12 | 6.39 | lingual gyrus |
| -22 | 30 | 50 | 6.19 | superior frontal gyrus |
| -24 | 22 | 44 | 5.96 | superior frontal gyrus |
| -14 | 32 | 56 | 6.08 | superior frontal gyrus |
| 24 | 22 | 54 | 5.98 | superior frontal gyrus |
| 12 | -52 | 8 | 5.75 | cuneus |
| 8 | -46 | 4 | 4.54 | cuneus |
| 28 | -40 | -10 | 5.50 | parahippocampal cortex |
| 24 | 12 | 66 | 5.15 | superior frontal gyrus |
| 26 | -32 | -14 | 4.38 | parahippocampal cortex |
| 36 | -34 | -14 | 4.24 | parahippocampal cortex |
